# Nutritionist’s Variation in Counseling Style and the Effect on Weight Change of Patients Attending a Community Based Lifestyle Modification Program

**DOI:** 10.3390/ijerph7020413

**Published:** 2010-02-02

**Authors:** Kris Y. W. Lok, Ruth S. M. Chan, Mandy M. M. Sea, Jean Woo

**Affiliations:** 1 Department of Medicine & Therapeutics, Chinese University of Hong Kong, Hong Kong, China; E-Mails: ruthchansm@cuhk.edu.hk (R.S.M.C.); jeanwoowong@cuhk.edu.hk (J.W.); 2 Centre for Nutritional Studies, Chinese University of Hong Kong, Hong Kong, China; E-Mail: mandysea@cuhk.edu.hk

**Keywords:** dietary adherence, lifestyle modification, weight management, obesity

## Abstract

Information concerning the nature of nutritionist-patient relationships is very limited. This qualitative and quantitative study examined nutritionist’s skills, attributes, and beliefs towards nutrition counseling during a lifestyle modification intervention program, and whether this affected the patient’s weight outcome. 24 nutrition consultations were observed during the program and the nutritionists were interviewed for their perception on practice (n = 4). A statistically significant difference was observed between the nutritionists in regard to patient’s weight change after adjustment for age and baseline weight (p < 0.001). Key nutritionist skills identified that influenced weight outcome were meticulous investigation of the underlying obesity cause, identification of the subject’s stage of change, and psychological support.

## 1. Introduction

Obesity is posing a growing threat to public health throughout the world and places extra burden on health costs. According to the Population Health Survey conducted in 2004 in Hong Kong, 38.8% of the population were overweight and 21% were obese [1]. This is a major cause of concern as obesity is often related to other health risks, such as cancer, heart disease, diabetes, dyslipidemia, hypertension, and stroke [1]. While prevention is clearly important, effective management of those with obesity continues to be a public health challenge.

Common management options consist of lifestyle modifications in terms of diet and physical activity, pharmacological methods, and surgery for the morbidly obese [2]. It has been proposed that a weight management and treatment algorithm should consist of diet, exercise, and behavioral therapy [3]. As a result, numerous commercial and proprietary weight loss programs have been introduced [3]. Studies have shown that the inclusion of a component of behavioral modification is crucial to the success of most programs [2,4].

Most commercial weight loss programs in Hong Kong offer a range of options from very low calorie exchange diets, meal replacement products, to supplements or unbalanced meals. Often these may be unaffordable for many people or carry claims of unrealistic weight loss. The Lifestyle Modification Program (LMP) examined in this study is a community based program in Hong Kong for those who are overweight or obese. It provides low calorie exchange diets and physical and behavioral weight control methods individualized by nutritionists during counseling sessions. The purpose of the LMP is to develop lifestyle behaviors that encourage increasing caloric expenditure while decreasing caloric intake with an emphasis on long term lifestyle and behavior change. Cognitive behavior therapy (CBT) is the strategy applied for behavior modification in the LMP [5]. CBT is derived from cognitive and behavioral psychological models of human behavior, which includes theories of normal and abnormal development, as well as theories of emotion and psychopathology. Five levels of influence were identified by McLeroy and colleagues for health related behaviors and conditions [6]. These levels include: (1) intrapersonal or individual factors; (2) interpersonal factors; (3) institutional or organizational factors; (4) community factors; and (5) public policy factors. The LMP highlights the importance of collaboration between clients and nutritionists to identify and understand the problems in terms of the relationship between thoughts, feelings, and behavior, and to find solutions. To test and re-test these solutions is important for cognitive and behavior changes. The LMP therefore focuses on exploring the intrapersonal and interpersonal factors that influence the client in order to make lifestyle changes. Intrapersonal factors, such as knowledge, attitudes, beliefs, and personality traits; and interpersonal factors, such as the social network and social support systems, are identified by the LMP nutritionists for cognitive and behavior changes [6]. A process of change may be involved, in which clients adjust their attitudes to the problems, consider the consequence of the problems, and assess self-confidence in relation to a possible change [7]. The LMP also highlights the use of a patient-centered approach in the counseling process. A patient-centered approach can be defined as involving the health professional trying to understand how the patient views their problems, and then working together on potential solutions in such a way that the patient feels they have developed their own realistic plans for change [8]. Five main domains are advocated in the patient-centered approach, which includes (i) exploring the patient’s main reason for the visit, their concerns, and need for information; (ii) seeking a comprehensive understanding of the patient’s personal, developmental, and family issues; (iii) finding a common understanding of what the problem is and mutually agreeing on a management plan; (iv) enhancing risk reduction and health promotion; and (v) enhancing the continuing relationship between the patient and the therapist [9,10].

As lifestyle modification is a complex process that involves both behavioral and cognitive changes [11], multiple theories are combined in the CBT of the LMP. These theories include firstly the Health Belief Model [12], which addresses the individual’s perceptions of the risk posed by a health problem (susceptibility and severity), the benefits of avoiding the risk, and factors influencing the decision to act (barriers, cues to action, and self-efficacy). Secondly, the Transtheoretical Model (TTM) [13], which describes an individual’s motivation and readiness to change a behavior. TTM involves 10 processes of change differentially applied during the five stages of change. The 10 processes of change are as follows: consciousness raising, self-liberation, social liberation, self re-evaluation, environmental re-evaluation, counter-conditioning, stimulus control, reinforcement management, dramatic relief, and helping relationships [13]. Thirdly, the Social Cognitive Theory [14], which describes the dynamic, ongoing process in which personal factors, environmental factors, and human behavior exert influence upon each other. And lastly, the Consumer Information Processing Theory [15], which postulates that information must not only be available but also believed to be useful and must be format-friendly to the consumer.

Patient education and counseling skills have been identified as key intervention strategies tackling obesity. Few studies have explored the effects of nutritionist’s views and practice on the outcomes of diet counseling. Predictors such as age and gender of individuals are not modifiable in order to potentially increase the chance of success on a weight loss program. However, variations in nutritionist’s counseling styles that may influence weight outcome can be modified. The present study aimed to explore the views of four nutritionists (A, B, C, D) and observed their practice and relationship with patients attending a community based LMP on lifestyle and behavior change, and whether this affected the outcome of LMP in terms of overall weight loss. Although the four nutritionists used the same dietary assessment tool and meal planning manual during nutrition counseling, we expected that there were differences in their counseling styles and interactions with clients and this staff variation was one of the factors affecting the weight outcome of LMP.

## 2. Methods

### 2.1. The LMP Components

The Lifestyle Modification Program (LMP) is carried out at the Centre for Nutritional Studies, The Chinese University of Hong Kong. The LMP design is adopted from the experience of a previous study [2]. As mentioned earlier, lifestyle modification involves both behavioral and cognitive changes [11]. The LMP is developed based on the CBT concept and multiple theories.

Upon enrollment into the program, all individuals undergo a comprehensive health and dietary assessment; each individual completes an initial nutrition assessment with a registered nutritionist. During the initial assessment, the nutritionist carries out a complete behavioral assessment and collects important information, such as the client’s history of health problems, current eating and lifestyle patterns, specific eating-related behaviors, knowledge of risks associated with current eating patterns, concerns and feelings about specific lifestyle changes (perceived susceptibility and perceived severity), past experiences and barriers to change in dietary intake and lifestyle (perceived barriers), reasons for changing current eating patterns (cues to action), and readiness to change (stage of change). The nutritionist also discusses with the client the expected duration and specific dietary and lifestyle advice to achieve a desirable weight status. Thereafter, an exercise instructor designs individualized cardiovascular and resistance exercises to perform at home. On completion of the nutrition assessment and exercise advice, clients are encouraged to attend weekly 15 to 20 minute counseling sessions with the same nutritionist and exercise instructor.

During the follow up counseling, the nutritionists review the client’s diet compliance, exercise adherence, and progression. The nutritionists review compulsory food records to ensure nutritional adequacy of the diet and offer recommendations for controlling caloric intake. The dietary component and portion sizes of the program are based on American Dietetic Association exchange lists, with 20% caloric restriction as appropriate to the individual [16]. A varied balanced diet with an emphasis on fruit and vegetables, and low-fat, low-glycemic index, and low calorific products in appropriate portions are encouraged. Clients are encouraged to increase daily activity, accumulating 30 to 60 minutes of cardiovascular activity one to five days a week. Clients can repeat these exercise consultations as often as they feel necessary. In addition to reviewing the client’s diet compliance and exercise adherence, the nutritionists encourage the clients to share any personal and environmental barriers to lifestyle change. The nutritionists aim to assess the client’s feelings with the dietary advice, provide ongoing support and encouragement, and affirm the client’s efforts and achievements. To minimize the barriers to lifestyle changes, each client is provided with a booklet for portion size exchange and tips for eating out. The nutritionists also encourage the clients to bring new food products and restaurant menus to discuss and explore healthy choices, and to send any enquiries to their nutritionist to exchange case management skills and updated information on nutrition, lifestyle and health.

### 2.2. Training of Nutritionists

The LMP training was developed and conducted in several steps. First, a nutritionist manager trained the nutritionists in CBT concept, multiple theories of behavioral change, and the LMP practice. The training consisted of two one-week training sessions. The first week included an introductory training, in which the trainees shadowed the nutritionist manager; the second week consisted of training the nutritionists mostly on knowledge and dietary counseling skills using the CBT concept and multiple behavioral change theories. Basic CBT and behavioral theories were specifically explained to each nutritionist working in the LMP. Each month, the nutritionists further participated in half-day follow up case discussions with the nutritionist manager to discuss initial experiences with the patients and to refresh their CBT knowledge and dietary counseling skills. Finally, they received on-demand feedback and advice on CBT-related issues by the base trainer, supervised until September 2002 when the actual study started. The demographics of the nutritionist participants were female fresh graduates with a Bachelor of Nutrition or Food Science related degree and were members of the British Nutrition Society as registered nutritionists. They had not undertaken any training in counseling and the language of counseling was conducted in Chinese.

### 2.3. Study Participants

1,501 participants were self-referred to the LMP between 2002 and 2006. Research participants were drawn from the Chinese University of Hong Kong, Centre for Nutritional Studies cohort (n = 645) between 2002 and 2006 who met the following inclusion and exclusion criteria. Inclusion criteria were: aged at least 18 years and less than 65 years, BMI ≥ 23 kg/m^2^, and enrollment of at least 12 weeks with completed weight measurements at baseline and at 12 weeks. Based on the World Health Organization’s criteria for Asians, the classifications of BMI of 23–24.9 kg/m^2^ as overweight and BMI ≥ 25 kg/m^2^ as obese were used [17]. Each participant attended one session each week. Participants on any prescription medication were excluded. They were informed of the study when they attended their appointment, and recruited on a voluntary basis. No reward was given to the participants. Informed consent was obtained from all participants. The study was approved by the Ethics Committee of the Chinese University of Hong Kong.

### 2.4. Procedures and Data Analysis

Four nutritionists were based at the centre and subjects were randomly allocated based on first available appointment. The LMP had a structured approach with each subject attending a session once a week with the same nutritionist. The nutritionist manager (nutritionist A) was selected to enable comparison with the other three nutritionists (nutritionists B to D) whom received training as stated above. Upon receiving the client’s consent, the LMP counseling process was audio-taped and transcribed by the researcher. Themes relating to attributes of nutritionists were identified through coding of clinician’s and client’s data and case comparisons.

Individual interviews were conducted by two interviewers with a semi-structured interview guide to collect nutritionist’s views. Topics included weight management skills and techniques for obesity counseling according to CBT [11], and nutritionist’s view on practice and perceptions of what makes a good nutrition counselor. Recall of a recent encounter with an obese patient was used to ground the discussion. The interviews lasted 30 minutes. Each interview was recorded and transcribed verbatim. The transcripts were read twice by each interviewer. Key words and phrases in the transcripts were identified, compared and formed into main themes and subthemes using method described by Ryan and Bernard [18], which were discussed jointly together by the interviewers.

24 subjects (six per nutritionist) were approached to join the study and agreed to allow an observer to watch the nutritionist-client communication in one session. The observation instrument is based on the themes set in the evidence that health professionals need to achieve longer term weight control, and is based on the recommendations for improved training in obesity management for all health professionals [8]. A final sample of 24 subjects were used as no new information emerged from the last few observation sessions of each round indicating that saturation had occurred [19].

### 2.5. Statistical Analysis

Weight, height, BMI, blood pressure, body fat % and waist and hip circumference were measured at baseline, whereas weight was measured again at 12 weeks. Changes from baseline were calculated. After normality was checked and log transformation was applied to baseline weight, six outliers of weight change were excluded from the study. A student’s *t* test was used to examine gender differences in age and baseline characteristics. Paired *t* test was used to examine the weight change between baseline and 12 weeks. ANOVA was used to examine the difference in age, baseline characteristics and weight change between the groups that each nutritionist was assigned. ANCOVA was used to examine the effect of nutritionist variation on weight. Men and women were analyzed separately. Weight change was entered as dependent variable. The fixed factor was nutritionist whereas covariates included age and baseline weight. Nutritionist A was used as reference category in the contrast test. SPSS version 13.0 was applied and all tests are two sided with the significance level set at 0.05.

## 3. Results

### 3.1. Participant Characteristics

Characteristics of participants are presented in Table 1. Relative normal values of BMI, blood pressure, waist and hip circumference from the Population Health Survey (2003−2004), with a median age of 42 and 43 for females and males respectively [1], and the Heart Health Survey (2004/2005) by four adult age groups [20] were compared with the study participants. All anthropometric data and blood pressure data of the study participants were higher than the relative normal values, supporting that the study sample was overweight and obese.

### 3.2. Nutritionist Variation in Baseline Patient Population Profile

No significant differences were found in the gender distribution among patients of the four nutritionists. There were significant differences found in some baseline characteristics of female patients, but not male patients. These included weight (p = 0.001), BMI (p = 0.003), % body fat (p = 0.046), systolic blood pressure (p = 0.002) and hip circumference (p = 0.001). ANOVA was used to examine these differences among the four nutritionists; significant differences were found in patient’s weight, BMI, % fat, systolic blood pressure and hip circumference between nutritionists A & B’s and nutritionists A & C’s populations of patients, but no differences were found between nutritionists A & D’s and nutritionists C & D’s populations of patients (details not shown).

### 3.3. Effectiveness of the LMP

The mean weight change of all individuals completing the program is shown in Table 2. There were significant reductions in weight and BMI (p < 0.001) with an average of nine percent loss of initial body weight after 12 weeks on the LMP.

Subject’s age, baseline weight, gender, and staff variation were significantly associated with the weight change. Increasing age was significantly associated with a smaller weight change (r = 0.04, p = 0.001 in women; r = 0.09, p < 0.001 in men). Subjects with greater baseline weight (log transformed) showed greatest weight change (r = −11.14, p < 0.001 in women; r = −18.66, p < 0.001 in men). Women showed smaller weight reduction than men (*t* = 9.48, p < 0.001). When other variables were held constant, there was nutritionist variation in LMP performance (measured as weight outcome). Table 3 shows the effect of staff variation on weight change during the LMP. Nutritionist C showed significant difference from nutritionist A (p < 0.001) in women, and nutritionist D showed significant differences from A in both women and men (p < 0.001 in women; p < 0.01 in men), but there was no significant difference observed in patient’s weight change between nutritionist A and B .

### 3.4. Perceived Skills, Approach and Attitude of the Nutritionists on the LMP

Based on the data emerging from the interviews, all the themes and subthemes identified are shown in Table 4. Further qualitative analysis was performed to provide an insight explaining such differences through semi-structured interviews of individual nutritionists.

Nutritionist’s vision of what the LMP is was explored. Nutritionists A and B shared the same vision in “helping people achieve a healthy lifestyle including three components: diet, exercise and psychological health.” On the other hand, nutritionists C and D had the vision “to improve health status and lifestyle through provision of dietary advice and lifestyle modification advice” and lacked the importance of psychological aspects of the LMP.

The general view held by nutritionists on their role was to provide information, emotional support, and guidance. Nutritionists C and D understood that their role as a nutritionist was “a provider of information and of emotional support”. A and B also held this view but added that “…nutritionists are also a guidance provider to point out right and wrong and then to provide suggestions.”

Common themes emerged from all four nutritionists on the importance of establishing a good rapport and relationship with the patient. Nutritionists aimed to treat patients as a friend to show genuine empathy:

“…to test their acceptance level and attitude…for different patients we would use a different tone to show we care for them and enhance our relationship with patients. It is important not to interrupt patients, to ask questions, to show respect, and build up a rapport like a friendship” (Nutritionist A).

Professionalism was also seen as important to empathy and rapport as viewed by nutritionists A and B.

“…being firm and professional, to share personal matters to show understanding” (Nutritionist A) and illustrating a “strong tone and strictly pointing out the problem” (Nutritionist B).

There were differences in how to influence weight loss. Nutritionists A and B adopted a staged paradigm for such intervention to be matched to the stage of change of each individual, and that an understanding of this psychological (CBT theory) aspect was essential for sustaining weight loss during the intervention.

“…We need to capture the client’s attitude towards weight loss…use different strategies depending on the patient …discussing client’s motivation and assess readiness to change …and be flexible and use different strategies for different cases and provide ongoing support by follow up calls” (Nutritionist A).

“…need to adjust advice to patient’s current lifestyle…and discuss with clients about the readiness to change and their own responsibility” (Nutritionist B).

In contrast, nutritionists C and D placed more emphasis on empathy and rapport with the patient as their main strategy and lacked change statements.

Nutritionists A and B had a shared understanding of the importance of the nutritionist-patient relationship in helping patients find the underlying issues of their obesity and solutions through meticulous investigation. This was also validated by the independent observer, see Table 5.

“…sometimes we need to change roles and think from patient’s perspective as if we were in their situation…trust is an important issue, as is nutritionist-patient sharing to help tackle the problem together” (Nutritionist A).

“…as a counselor to remind patients to self-reflect on their problem and situation to help find solutions together” (Nutritionist B).

Nutritionists C and D emphasized empathy and rapport as essential skills in the treatment of obesity without extracting the key obstacles and psychological aspects that patients are experiencing with weight loss. Therefore the use of the psychological aspect of the stage of behavior change model was not observed with nutritionist C and D. Table 5 demonstrates comparable client-centered qualities in nutrition counseling.

## 4. Discussion

This study identified that predictors of the effectiveness of the Lifestyle Modification Program weight loss intervention over 12 weeks were client’s age, gender, and baseline weight. Males achieved significantly greater weight loss than women. These results concur with the results of previous studies [8,21,22], although one study consisted of a smaller sample (<100) that was followed for 3−12 months [19]. The predictors of age and gender are not modifiable in order to potentially increase the chance of success on a weight loss program. However, there were important variations in nutritionist’s counseling styles that may influence weight outcome for clients of the LMP.

The LMP demonstrated that subjects on the lifestyle intervention with a weekly follow up for 12 weeks lost an average of 9% of body weight. In accordance with other Western data, another weight management program in the Hong Kong Chinese population resulting in a 4%–6% reduction in body weight was associated with significant reductions in all cardiovascular risk factors [8].

All nutritionists understood counseling strategies such as the theory of empathy and establishing rapport with the client as their main strategies, but differences were observed in the translation into practice of CBT and other behavioural change theories. The communication skills of nutritionists A and B differed notably to nutritionists C and D; A and B used more complex communication skills and psychological support of the TTM in order to better motivate patients [13].

Lifestyle modification is a complex process, which involves both behavioural and cognitive changes [11]. Behavioral changes can be achieved by working together with the patient to investigate the underlying issues of obesity [23]. At the same time, before nutritionists can be successful with patient’s weight outcome, an important element is the nutritionist’s positive acceptance towards overweight patients to enable behavioral changes. This concurs with previous studies that show that a positive professional attitude towards obese individuals leads to positive experiences among health professionals specializing in obesity [24]. Another study found that dietician’s perceptions and attitudes may adversely affect the counsellor-client relationship and success of managing overweight clients. To enhance the quality of counseling, dieticians need to provide support and exhibit a caring attitude when interacting with clients who are overweight [25].

Nutritionists A and B held similar perceptions and attitudes about their role in obesity management, which may explain why no significant differences were found in weight outcomes. Nutritionists A and B demonstrated a comparable number of client-centred qualities in nutrition counseling (see Table 5). Therefore nutritionists need to be trained to conduct programs in the same way, as this can affect their relationship with clients and consequently affect weight outcome.

Nutritionist’s communication skills and constructive feedback are essential to deliver clear practical dietary and lifestyle advice towards a successful weight outcome as demonstrated in this study. Nutritionists A and B possessed a better questioning and commenting style response to patients *i.e.*, better communication skills and constructive feedback compared with nutritionists C and D. Nutritionists C and D were observed to be judgemental in their questioning style, which may explain the variations in the results of nutritionists A and C and nutritionists A and D. Evidence suggests some form of positive feedback should always be given; health professionals can view feedback as detective work, and as a useful tool for guiding patients to generate their own solutions to barriers and new problems. This approach avoids the common scenario of health professionals telling patients what they think the patient should do [8].

Client-centered practice was understood by nutritionists in this study and remains an effective strategy to conduct consultations with patients. The core qualities nutritionists agreed upon were unconditional acceptance, genuineness, and empathy, all highly important to achieve rapport with patients during consultations. This has been increasingly advocated and incorporated into practice [26]. Recent data indicate that the patient-centered counseling model enhances long term dietary adherence. This model facilitates change by assessing patient needs and subsequently tailoring the intervention to the patient’s stage in the process of change, personal goals, and unique challenges [27]. Patient centered counseling involves a four-step process including: a brief, but thorough assessment of the patient’s current stage of change relative to general or specific aspects of dietary behavior; advice with respect to the need to change specific eating behaviors; assistance in changing such behaviors; and arranging follow up to monitor how plans are proceeding [27]. The skilled nutritionist’s task is to translate theory into practice; recognizing patient’s health beliefs and stage of change and motivating them by providing psychological support. The skills required include effective communication and investigation to resolve issues underlying obesity.

A limitation to the current study is that it is not a randomized control trial, so no control group was used. Qualitative evaluation of counseling assessment is dependent on who performs the analysis, which can lead to different interpretations. Observational assessment was conducted by an observer and only four nutritionists were observed. The quality of observational data is highly dependent on the skill, training, and competence of the observer. A major concern about the validity and reliability of observational data concerns the effects of the observer on the subject being observed. People may behave differently when they know they are being observed compared to how they behave if they are not aware of being observed [28]. Clearly, the depth, detail, and sensitivity of data will vary according to assessment tool used, and a scoring system would increase validity. Client’s perceptions of the treatment and of the nutritionists have not been evaluated here. This would potentially have been a rich source of qualitative information that may have validated the independent observer’s ratings, and allowed a linking of therapeutic style to weight loss. Participants paid a fee to join and stay in the LMP program, and those who dropped out were excluded; thus the sample may be biased towards more successful individuals. Although the sample is biased towards motivated and successful individuals, the main focus was not to make generalizations about the population from which the sample was drawn, but rather to explore the range of skills and attitude of the nutritionists towards successful obesity management outcomes, therefore the research design was appropriate.

This study included the relatively short follow up period of subjects over 12 weeks, and the effect of long term weight maintenance is unknown. We did not formally assess compliance of exercise with our subjects nor their dietary intake, both important factors influencing their weight change. Other client factors could have also affected weight loss, such as stage of change, depression, eating disorder symptoms, and weight loss goals. These factors need to be taken into account as possibilities as well as the qualitative evaluation found in the therapeutic style of the nutritionists. These issues are clearly of great importance and represent limitations to our study.

## 5. Conclusions

This study highlights the need for increased training opportunities and setting protocols on obesity treatment approach. This will help standardize nutritionist’s approaches towards assessment and behavioural management strategies in tackling the underlying issues of obesity. Counseling skills using the client-centered patient model should be emphasized in continued education programs of nutritionists and allied health professionals specializing in obesity. More observational assessment is needed to help strengthen training and skills of practitioners.

## Figures and Tables

**Table 1.** Subject characteristics at program enrollment.

**Subject *value*^1^ Characteristics**	**Female (n = 500)**	Relative normal values	**Male (n = 145)**	Relative normal values	***P***
Age (years ± SD)	40.8 ± 9.9	-	39.7 ± 10.2	-	0.241
**BMI** (kg/m^2^ ± SD)	28.8 ± 9.9	22.3	30.7 ± 3.7	24.0	0.000
*% variation*	*+2.9%*		*+2.8%*		
Body Fat (% ± SD)	39.3 ± 5.6	-	30.8 ± 4.9	-	0.000
**Systolic Blood Pressure** (mmHg ± SD)	128.3 ± 17.3	122.9 ± 21.6*	134.8 ± 16.8	129.6 ± 18.8*	0.000
*% variation*	*+4.4%*		*+4.0%*		
**Diastolic Blood Pressure** (mmHg ± SD)	77.0 ± 10.8	74.8 ± 12.2*	82.3 ± 11.3	77.9 ± 12.5*	0.000
*% variation*	*+2.9%*		*+5.6%*		

**Waist circumference** (cm ± SD)	88.1 ± 8.3	78.2	101.0 ± 8.7	85.1	0.000
*% variation*	*+12.7%*		*+18.7%*		
**Hip circumference** (cm ± SD)	104.3 ± 7.3	92.9	107.3 ± 6.6	93.5	0.000
*% variation*	*+12.3%*		*+14.8%*		

1 difference by Student’s t test,

* Source: Population Health Survey 2003–2004^1^.

**Table 2.** Comparison of changes in weight and clinical outcomes before and after the LMP.

Female (n = 500)					
**Parameters**	Baseline (Mean ± SD)	12 weeks (Mean ± SD)	% change	*P-value*^1^	
Weight (kg)	70.6 ± 9.9	64.2 ± 9.5	9.2	<0.001	
BMI (kg/m^2^)	28.8 ± 3.2	26.2 ± 3.1	9.2	<0.001	

Male (n = 145)					
**Parameters**	Baseline (Mean ± SD)	12 weeks (Mean ± SD)	% change	*P-value*^1^	*P-value*^2^
Weight (kg)	88.7 ± 10.9	79.7 ± 10.1	10.1	<0.001	<0.001
BMI (kg/m^2^)	30.7 ± 3.7	27.6 ± 3.4	10.1	<0.001	<0.001

1 pre-post difference by paired *t* test;

2 gender difference by Student’s *t* test.

**Table 3.** Effect of nutritionist variation on weight change during the LMP by gender.

**Gender**	**Nutritionist**				***P* value**^1^
	A	B	C	D	
Female					<0.001
n	270	56	98	76	
Estimated mean ± SE	−6.9 ± 0.1	−6.5 ± 0.3^ns^	−5.9 ± 0.2^***^	−5.4 ± 0.3^***^	
Male					0.026
n	84	23	19	19	
Estimated mean ± SE	−9.4 ± 0.3	−8.9 ± 0.5 ^ns^	−8.7 ± 0.6 ^ns^	−7.5 ± 0.6^**^	

1 difference by ANCOVA adjusted for age and baseline weight;

ns, non significant;

**, p < 0.01;

***, p < 0.001 (nutritionist A as reference category).

**Table 4.** Themes identified by researchers from analysis of transcribed interviews.

**Themes**	**Subthemes**
Perceived vision of the LMP	On helping people achieve a healthy lifestyle and provide dietary advice On providing psychological support
Role in the LMP	Nutritionist being an information provider Nutritionist being a psychological supporter in the LMP
Attitude towards patients	Good rapport and genuine empathy Maintain professionalism
Strategy to tackle weight loss	Adoption of CBT with ongoing psychological support Empathy and good rapport strategy
Counseling skills	Meticulous investigation of the underlying cause of obesity Empathy and rapport

**Table 5.** Observational evaluation of nutritionists using 24 client interviews.

	Nutritionist			
	A	B	C	D
Help client understand the problem and suggestions	✓	✓	✓	✓
Non-responsive to client effort	x	x	x	✓
Responsive to client preference	x	x	✓	✓
Responsive to client worry	✓	x	x	✓
Judgmental questioning style	x	x	✓	✓
Good questioning style	✓	✓	✓	x
Good commenting style	✓	✓	x	x
Work out solution together	✓	✓	✓	✓
Praise client effort	✓	✓	✓	✓
Cognitive restructuring	✓	✓	x	x
Goal setting – identify barrier	x	x	✓	x
Goal setting – help client set realistic goal	✓	✓	x	x
Considerate	✓	✓	✓	x
Encouragement	✓	✓	✓	✓
Acceptance, supportive	✓	✓	✓	x
